# Synthesis, crystal structure and Hirshfeld surface analysis of the ortho­rhom­bic polymorph of 4-bromo-*N*-(4-bromo­benzyl­idene)aniline

**DOI:** 10.1107/S2056989023001111

**Published:** 2023-02-09

**Authors:** A. Subashini, K. Ramamurthi, R. Ramesh Babu, Reji Philip, Helen Stoeckli-Evans

**Affiliations:** a PG and Research Department of Physics, Arts and Science College, Tiruchirappalli - 620 005, India; b Crystal Growth and Thin Film Laboratory, Department of Physics, Bharathidasan University, Tiruchirappalli - 620 024, India; c Ultrafast and Nonlinear Optics Laboratory, Raman Research Institute, C. V. Raman Avenue, Sadashivanagar, Bangalore 560 080, India; d Institute of Physics, University of Neuchâtel, rue Emile-Argand 11, 2000 Neuchâtel, Switzerland; University of Aberdeen, United Kingdom

**Keywords:** crystal structure, *N*-benzyl­ideneaniline, polymorphism, Hirshfeld surface, fluorescence, two-photon absorption

## Abstract

The crystal structure of the title compound, C_13_H_9_Br_2_N [systematic name: (*E*)-*N*,1-bis­(4-bromo­phen­yl)methanimine], is a second polymorph (Form II) crystallizing in the ortho­rhom­bic space group *Pccn*. The first polymorph (Form I) crystallizes in the monoclinic space group *P*2_1_/*c* [Bernstein & Izak (1975 ▸). *J. Cryst. Mol. Struct.* **5**, 257–266; Marin *et al.* (2013 ▸). *J. Mol. Struct.* **1049**, 377–385]. The mol­ecule is disordered about an inversion center situated in the middle of the C=N bond, similar to the situation in the monoclinic polymorph: the C=N bond length is 1.243 (7) Å. In the crystal, mol­ecules stack along the *b-*axis direction and are linked by C—H⋯π inter­actions. The inter­atomic contacts in the crystal for both polymorphs were studied by Hirshfeld surface analysis and have notable differences. The solid-state fluorescence spectrum of Form II shows an emission peak at *ca* 469 nm. The two-photon absorption coefficient measured from the open aperture *Z*-scan technique is 1.3 × 10 ^−11^ m W^−1^, hence, Form II shows optical limiting behaviour.

## Abstract 2

The crystal structure of the ortho­rhom­bic polymorph of *N*-(4-bromo­benzyl­idene)-4-bromo­aniline, C_13_H_9_Br_2_N, is reported for the first time. The mol­ecule is disordered about a crystallographic inversion center.

## 1. Chemical context

Many compounds possess the ability to form polymorphs (Rolf, 2006 ▸; Caira, 2017 ▸) and polymorphism and disorder are well established in *N*-benzyl­ideneaniline compounds. Homo-disubstituted benzyl­ideneaniline compounds such as *N*-(4-chloro­benzyl­idene)-4-chloro­aniline (Bernstein & Schmidt, 1972 ▸; Bernstein & Izak, 1976 ▸) and *N*-(4-methyl­benzyl­idene)-4-methyl­aniline (Bar & Bernstein, 1977 ▸, 1982 ▸; Bernstein & Izak,1976 ▸) exist in dimorphic and trimorphic forms, respectively. Bar & Bernstein (1983 ▸) reported a detailed description of three types of disorder in *N*-benzyl­ideneanilines, *viz*., positional, orientational and substitutional. These various types of disorder have been discussed in relation to hetero-disubstituted 4-*X*-*N*-(4′-nitro­benzl­idene)anilines (where *X* = H, F, Cl, Br, CH_3_, CH_3_O, OH) by Leela *et al.* (2020 ▸ and references therein). 
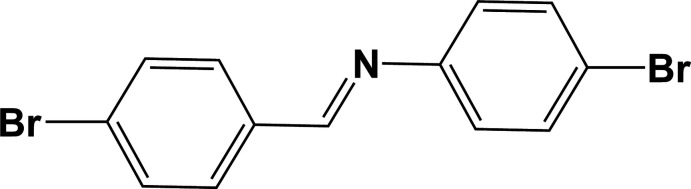


The crystal structure of the monoclinic polymorph of the title compound, *N*-4-bromo­benzyl­idene)-4-bromo­aniline, C_13_H_9_Br_2_N (Form I) has been reported by Bernstein & Izak (1975 ▸) and later by Marin *et al.* (2013 ▸). Bernstein & Hagler (1979 ▸) stated that they did not observe polymorphism for this compound. However, Marin *et al.* (2013 ▸) reported that a second polymorph could appear during thermal treatment.

In the present study the homo-disubstituted title compound was synthesized using the procedure described by Bernstein & Izak (1975 ▸). Single crystals were grown by slow evaporation of a solution in either ethanol or a mixture of methanol/chloro­form (1:1). Crystals from both essays proved to be those of the ortho­rhom­bic polymorph – Form II.

## 2. Structural commentary

**Figure 1:**
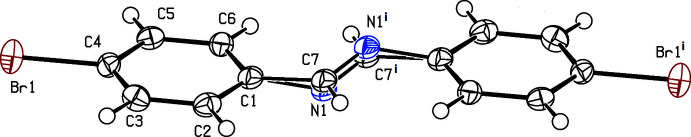
A view of the mol­ecular structure of Form II of the title compound, with atom labelling. Displacement ellipsoids are drawn at the 50% probability level. Symmetry code: (i) −*x*, −*y*, −*z*.

The title compound crystallizes in the ortho­rhom­bic space group *Pccn* (Form II). It exhibits disorder about a crystallographic inversion center situated on the middle of the C7=N1 bond, as illustrated in Fig. 1 ▸. This arrangement is similar to that observed for the monoclinic polymorph (Bernstein & Izak, 1975 ▸; Marin *et al.*, 2013 ▸) and for the ortho­rhom­bic and monoclinic polymorphs of the chloro-disubstituted *N*-benzyl­ideneaniline (Bernstein & Izak, 1976 ▸; Bernstein & Schmidt, 1972 ▸).

Atoms C7, H7 and N1 were refined with occupancies of 0.5 each. The C=N bond length is 1.243 (7) Å, and the benzene rings are co-planar by symmetry. These geometrical parameters are similar to those observed for Form I, the monoclinic polymorph (Marin *et al.*, 2013 ▸).

A search of the Cambridge Structural Database [CSD, V5.43, last update November 2022; Groom *et al.*, (2016 ▸)] for *N*-benzyl­ideneanilines with no *ortho*-substituents on the aromatic rings, no errors, no polymerics, no ions or disorder, for organics only and *R* < 0.05 gave 220 hits. An analysis of the geometry of these 220 compounds (*Mercury*; Macrae *et al.*, 2020 ▸) indicated that the C=N bond length varies from 1.1775 to 2.202 Å, with a mean value of 1.269 Å (mean deviation of 0.009 Å). Hence, the value observed for Form II is significantly shorter at 1.243 (7) Å, while that for Form I is close to this average at 1.257 (2) Å. The bond lengths N1—C1_ar_ and C7—C1_ar_ have mean values of 1.420 (mean deviation of 0.006 Å) and 1.462 Å (mean deviation of 0.008 Å), respectively. The values observed for Form I are the same at 1.439 (3) Å, and for Form II they are also the same at 1.45 (2) Å, owing to the fact that both polymorphs are disordered about an inversion centre.

## 3. Supra­molecular features

**Figure 2:**
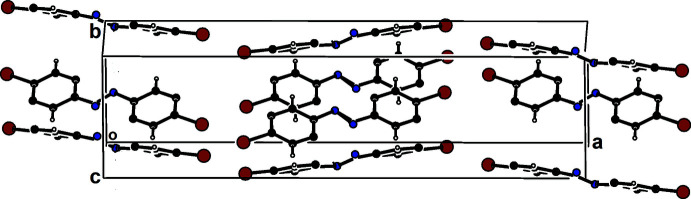
A view along the *c-*axis of the crystal packing of Form II of the title compound.

In the crystal, the mol­ecules are linked by C—H⋯π inter­actions (Table 1 ▸), forming columns stacking along the *b*-axis direction, as shown in Fig. 2 ▸.

## 4. Hirshfeld surface analyses and two-dimensional fingerprint plots for Form I and Form II

The Hirshfeld surface analysis (Spackman & Jayatilaka, 2009 ▸) was performed and the associated two-dimensional fingerprint plots (McKinnon *et al.*, 2007 ▸) were generated with *CrystalExplorer17* (Spackman *et al.*, 2021 ▸) following the protocol of Tan *et al.* (2019 ▸). The Hirshfeld surface is colour-mapped with the normalized contact distance, *d*_norm_, from red (distances shorter than the sum of the van der Waals radii) through white to blue (distances longer than the sum of the van der Waals radii).

**Figure 3:**
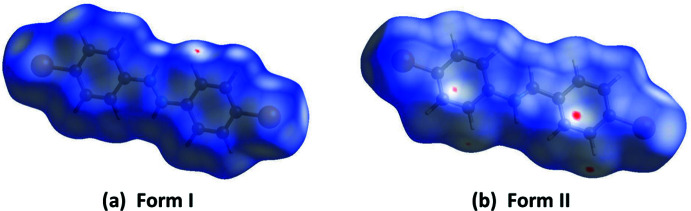
(*a*) The Hirshfeld surface of Form I, mapped over *d*_norm_ in the colour range −0.0139 to 0.7999 a.u. and (*b*) the Hirshfeld surface of Form II, mapped over *d*_norm_ in the colour range −0.0329 to 1.0662 a.u.

The Hirshfeld surfaces of Form I and Form II mapped over *d*_norm_, are given in Fig. 3 ▸. The faint red spots indicate that short contacts are significant in the crystal packing of both compounds.

**Figure 4:**
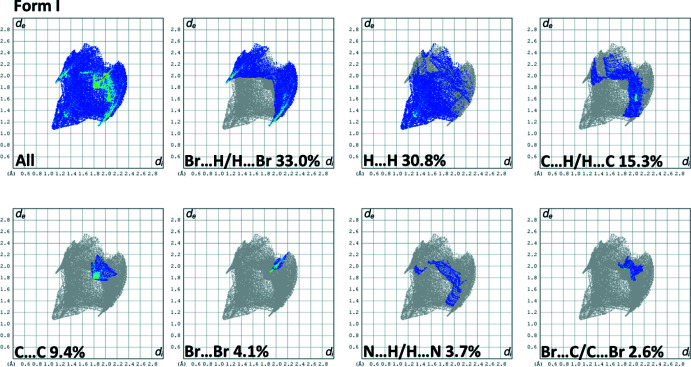
(*a*) The full two-dimensional fingerprint plot for Form I, and those delineated into Br⋯H/H⋯Br, H⋯H, C⋯H/H⋯C, C⋯C, Br⋯Br, N⋯H/H⋯N and Br⋯C/C⋯Br and contacts.

The full two-dimensional fingerprint plots for Form I and Form II are given in Figs. 4 ▸ and 5 ▸, respectively. There it can be seen that the relative contributions of the various inter­atomic contacts in the two polymorphs are significantly different (Table 2 ▸). For example, the C⋯H/H⋯C contacts have a major contribution (36.4%) in Form II whereas the same contact type is only 15.3% in Form I. This trend is reversed for the Br⋯H/H⋯Br contacts, which make a major contribution (33.0%) in Form I but less, 24.9%, in Form II, and for the H⋯H contacts which are greater in Form I (30.8%) compared to Form II (21.2%). The halogen⋯halogen inter­actions at the *para-*positions of the aromatic rings are more important in Form II (9.7%) compared to Form I (4.1%). Similar values of relative percentage are observed for the Br⋯C/C⋯Br contacts in Form I (2.6%) and Form II (2.3%). There are significant C⋯C inter­molecular contacts observed for Form I (9.4%) but they are very weak (0.1%) in Form II. In contrast, the N⋯H/H⋯N contacts observed for Form II (5.4) are stronger than those observed for Form I (3.7%).

## 5. Thermal properties

**Figure 6:**
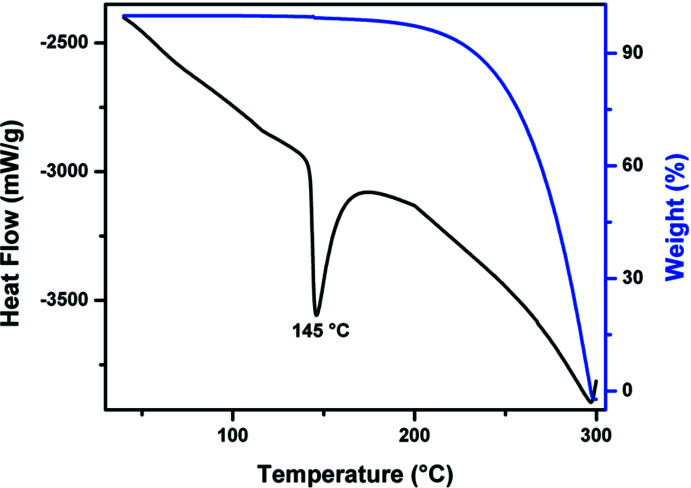
The TGA (blue) and DTA (black) curves for Form II.

Thermogravimetric (TGA) and differential thermal (DTA) analyses were recorded using an S·T.A. − 1500 Simultaneous Thermo Analytical system in the temperature region 30–300°C at a heating rate of 10 K min^−1^ under a nitro­gen atmosphere and alumina (Al_2_O_3_) was used as the reference material. The thermogravimetric and differential thermal analyses of Form II are shown in Fig. 6 ▸. In the DTA curve the sharp endothermic peak observed at *ca.* 145 °C corresponds to the melting point and indicates that there is no decomposition before melting. A single-stage weight loss is indicated in the TGA curve. At *ca* 300°C almost the entire mass of the sample is lost, thus indicating the occurrence of bulk decomposition.

## 6. Fluorescence spectra

**Figure 7:**
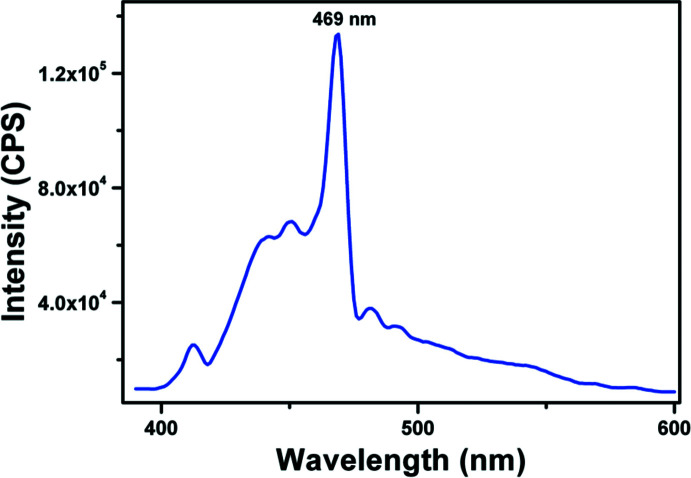
The solid-state fluorescence emission spectrum of Form II.

The fluorescence emission spectrum of Form II at room temperature was recorded using a Horiba Jobin Yuon FLUOROLOG-FL3-11 spectro­fluoro­meter in the wavelength range of 375–600 nm (Fig. 7 ▸). A powdered sample of Form II was excited at 375 nm and an emission peak was observed at 469 nm (blue colour) due to the presence of the aromatic rings (Lakowicz, 2006 ▸). For Form I, an emission peak was reported at 414 nm (weak violet colour) when excited at 278 nm (Marin *et al.*, 2013 ▸).

## 7. Z-scan studies

Employing the open aperture *Z*-scan technique the third order non-linear optical property of Form II was studied: the experimental setup has been described previously (Subashini *et al.*, 2013*a* ▸,*b* ▸). A sample of Form II was dissolved in chloro­form, and 5 ns laser pulses at 532 nm were used for excitation. The optical density of the Form II solution is low at 532 nm, and its linear transmission is high (85%). The laser pulse energy reaching the sample was 100 µJ. The open-aperture transmission normalized to the linear transmission of the sample (normalized transmittance) was then plotted against the sample position measured relative to the beam focus and the non-linear absorption was indicated by a smooth valley-shaped curve, which was symmetric about the focal (*Z* = 0) position. The Z scan of pure chloro­form was run separately to ensure that the solvent showed no non-linearity under the same experimental conditions.

**Figure 8:**
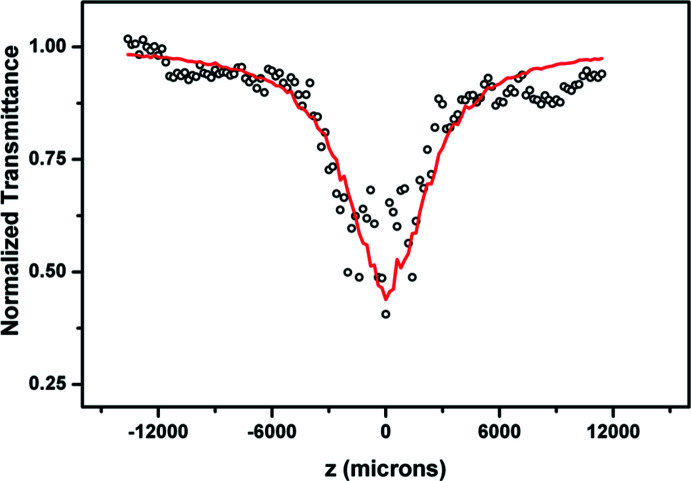
The open-aperture *Z* scan of Form II.

The results of the open-aperture Z scan of Form II are shown in Fig. 8 ▸. An increase in absorption is found as the laser intensity is increased, thus indicating the non-linear optical absorption and the optical limiting behaviour of Form II. Since the linear transmission of the sample is ∼85%, the observed non-linear absorption might have contributions from genuine two-photon absorption (2PA), as well as from excited state absorption (ESA). For further details of the data analysis, see supporting information.

## 8. Synthesis, crystallization and spectroscopic analyses

The title compound was synthesized by the condensation reaction of 4-bromo­benzaldehyde with 4-bromo­aniline in an equimolar ratio: the reactants were dissolved in ethanol and refluxed for 6 h at 363 K, then cooled to room temperature. The precipitated product was purified by repeated recrystallization using ethanol as solvent. Colourless crystals of the title compound were grown at room temperature by slow evaporation of a solution in either ethanol or a mixture of methanol/chloro­form (1:1).

**Figure 9:**
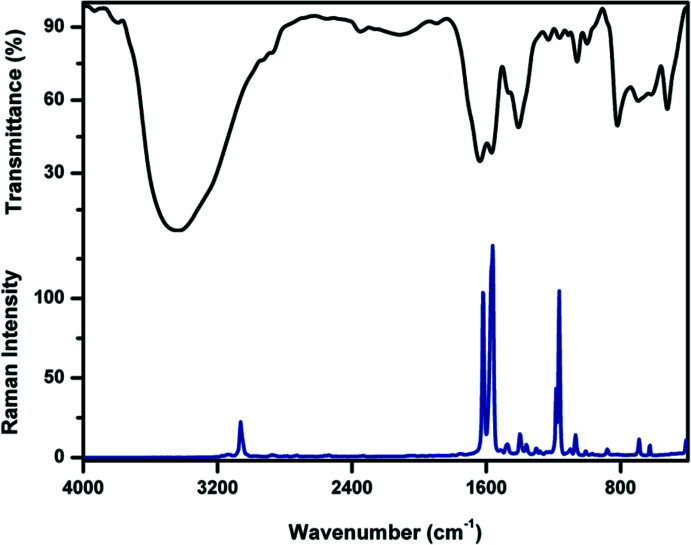
The FT–IR (black) and FT–Raman (blue) spectra of Form II.

The solid-state Fourier Transform Infrared (FT–IR) and Fourier Transform Raman (FT–Raman) spectra at room temperature (Fig. 9 ▸) were recorded using a Perkin Elmer grating infrared spectrophotometer (KBr pellet technique) and a Varian FT-Raman spectrometer, respectively, in the wave number range of 400–4000 cm^−1^ (Fig. 11). The C=N stretching vibration is observed as a strong band at 1630 cm^−1^ (IR) and at 1615 cm^−1^ (Raman) (Silverstein *et al.*, 2005 ▸). For Form I, the C=N stretching vibration was reported at 1620 cm^−1^ (Marin *et al.*, 2013 ▸). In the IR spectrum, the aromatic C=C stretching vibrations are observed at 1468 and 1562 cm^−1^, and at 1474 and 1558 cm^−1^ in the Raman spectrum. In the IR spectrum, the aromatic C—H in-plane bending modes are observed at 1059, 1111, 1163 and 1230 cm^−1^ whereas the out-of-plane bending modes appear at 819 and 1000 cm^−1^ (Yoshino *et al.*, 2013 ▸). In the Raman spectrum, the corresponding in-plane bending modes are observed at 1008, 1067, 1098, 1165 and 1183 cm^−1^ whereas the out-of-plane bending mode is observed at 879 cm^−1^. In the FT–IR spectrum, the band at 521 cm^−1^ is due to the aromatic C—Br stretching vibration.

The ^1^H and ^13^C NMR data for Form II in chloro­form-D (CDCl_3_) using tetra­methyl­silane as the inter­nal standard were recorded employing a Bruker AC 400-NMR spectrometer.

The ^1^H NMR spectrum (illustrated in Fig. S1 of the supporting information) exhibits five proton signals. The intense proton signal appearing at δ = 8.364 ppm is attributed to the imine group. The peaks at 7.48–7.52 and 7.59–7.61 ppm are due to the aromatic ring proton signals of the bromo­benzaldehyde moiety and the peaks at 7.07–7.09 and 7.74–7.76 ppm are due to the aromatic ring proton signals of the bromo­aniline moiety.

In the ^13^C NMR spectrum (illustrated in Fig. S2 of the supporting information) the peak at 76.46–77.40 ppm corres­ponds to the carbon atom of CDCl_3_. A peak corresponding to the imine carbon atom is observed at δ = 159.26 ppm. The peaks at 119.60, 122.54, 134.88 and 150.54 ppm correspond to the phenyl carbon-atom signals of the bromo­aniline moiety and the peaks at 126.19, 126.24, 130.19, 130.24, 132.09, 132.13, 132.24 and 132.29 ppm are due to phenyl carbon-atom signals of the bromo­benzaldehyde moiety.

## 9. Refinement details

Crystal data, data collection and structure refinement details are summarized in Table 3 ▸. The atoms N1, C7, and H7 were refined with occupancies of 0.5 each. The C-bound H atoms were included in calculated positions (C—H = 0.95 Å) and refined as riding atoms with *U*_iso_(H) = 1.2*U*_eq_(C).

## Supplementary Material

Crystal structure: contains datablock(s) I, global. DOI: 10.1107/S2056989023001111/hb8048sup1.cif

Structure factors: contains datablock(s) I. DOI: 10.1107/S2056989023001111/hb8048Isup2.hkl

Fig. S1: 1H NMR spectrum of From II. DOI: 10.1107/S2056989023001111/hb8048sup3.tif

Fig. S2: 13C NMR spectrum of Form II. DOI: 10.1107/S2056989023001111/hb8048sup4.tif

Data treatment for the Z-scan. DOI: 10.1107/S2056989023001111/hb8048sup5.pdf

Supporting information file. DOI: 10.1107/S2056989023001111/hb8048Isup6.cml

CCDC reference: 1583365

Additional supporting information:  crystallographic information; 3D view; checkCIF report

## Figures and Tables

**Figure 5:**
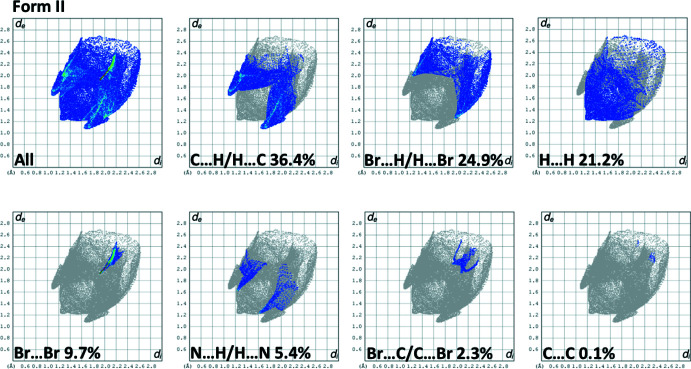
(*a*) The full two-dimensional fingerprint plot for Form II, and those delineated into C⋯H/H⋯C, Br⋯H/H⋯Br, H⋯H, Br⋯Br, N⋯H/H⋯N, Br⋯C/C⋯Br and C⋯C contacts.

**Table 1:** Hydrogen-bond geometry (Å, °) *Cg*1 is the centroid of the C1–C6 ring.

*D*—H⋯*A*	*D*—H	H⋯*A*	*D*⋯*A*	*D*—H⋯*A*
C2—H2⋯*Cg*1^i^	0.95	2.81	3.535 (3)	134
C5—H5⋯*Cg*1^ii^	0.95	2.78	3.494 (3)	133

Symmetry codes: (i) 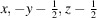 ; (ii) 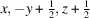 .

**Table 2:** The relative contributions of the various inter­atomic contacts (Å) in the crystal structures of Form I^*a*^ and Form II

Contact	Form I^*a*^	Form II
	%	%
H⋯H	30.8	21.2
C⋯H/H⋯C	15.3	36.4
N⋯H/H⋯N	3.7	5.4
Br⋯H/H⋯Br	33.0	24.9
C⋯C	9.4	0.1
Br⋯C/C⋯Br	2.6	2.3
Br⋯Br	4.1	9.7

Note: (*a*) Marin *et al.* (2013 ▸).

**Table 3:** Experimental details

Crystal data	
Chemical formula	C_13_H_9_Br_2_N
*M*_r_	339.03
Crystal system, space group	Orthorhombic, *P* *c* *c* *n*
Temperature (K)	173
*a*, *b*, *c* (Å)	27.4096 (19), 7.3301 (5), 5.9135 (3)
*V* (Å^3^)	1188.11 (13)
*Z*	4
Radiation type	Mo *K*α
μ (mm^−1^)	6.79
Crystal size (mm)	0.45 × 0.33 × 0.13

Data collection	
Diffractometer	STOE *IPDS* 2
Absorption correction	Multi-scan (*MULABS*; Spek, 2020 ▸)
*T*_min_, *T*_max_	0.411, 1.000
No. of measured, independent and observed [*I* > 2σ(*I*)] reflections	11334, 1125, 911
*R*_int_	0.075
(sin θ/λ)_max_ (Å^−1^)	0.609

Refinement	
*R*[*F* ^2^ > 2σ(*F* ^2^)], *wR*(*F* ^2^), *S*	0.037, 0.061, 1.09
No. of reflections	1125
No. of parameters	71
H-atom treatment	H-atom parameters constrained
Δρ_max_, Δρ_min_ (e Å^−3^)	0.34, −0.48

Computer programs: *X-AREA* and *X-RED32* (Stoe & Cie, 2009 ▸), *SHELXS97* (Sheldrick, 2008 ▸), *SHELXL2018*/3(Sheldrick, 2015 ▸), *PLATON* (Spek, 2020 ▸), *Mercury* (Macrae *et al.*, 2020 ▸) and *publCIF* (Westrip, 2010 ▸).

## supplementary crystallographic information

### Crystal data

**Table:**

C_13_H_9_Br_2_N	*D*_x_ = 1.895 Mg m^−^^3^
*M**_r_* = 339.03	Mo *K*α radiation, λ = 0.71073 Å
Orthorhombic, *P**c**c**n*	Cell parameters from 8006 reflections
*a* = 27.4096 (19) Å	θ = 1.5–26.1°
*b* = 7.3301 (5) Å	µ = 6.79 mm^−^^1^
*c* = 5.9135 (3) Å	*T* = 173 K
*V* = 1188.11 (13) Å^3^	Plate, colourless
*Z* = 4	0.45 × 0.33 × 0.13 mm
*F*(000) = 656	

### Data collection

**Table:**

STOE IPDS 2 diffractometer	1125 independent reflections
Radiation source: fine-focus sealed tube	911 reflections with *I* > 2σ(*I*)
Plane graphite monochromator	*R*_int_ = 0.075
φ + ω scans	θ_max_ = 25.7°, θ_min_ = 1.5°
Absorption correction: multi-scan (MULABS; Spek, 2020)	*h* = −33→33
*T*_min_ = 0.411, *T*_max_ = 1.000	*k* = −8→8
11334 measured reflections	*l* = −6→7

### Refinement

**Table:**

Refinement on *F*^2^	Secondary atom site location: difference Fourier map
Least-squares matrix: full	Hydrogen site location: inferred from neighbouring sites
*R*[*F*^2^ > 2σ(*F*^2^)] = 0.037	H-atom parameters constrained
*wR*(*F*^2^) = 0.061	*w* = 1/[σ^2^(*F*_o_^2^) + (0.0216*P*)^2^ + 1.2266*P*] where *P* = (*F*_o_^2^ + 2*F*_c_^2^)/3
*S* = 1.09	(Δ/σ)_max_ < 0.001
1125 reflections	Δρ_max_ = 0.34 e Å^−^^3^
71 parameters	Δρ_min_ = −0.48 e Å^−^^3^
0 restraints	Extinction correction: (SHELXL-2018/3; Sheldrick, 2015), Fc^*^=kFc[1+0.001xFc^2^λ^3^/sin(2θ)]^-1/4^
Primary atom site location: structure-invariant direct methods	Extinction coefficient: 0.0018 (2)

### Special details

**Table:**

Geometry. All esds (except the esd in the dihedral angle between two l.s. planes) are estimated using the full covariance matrix. The cell esds are taken into account individually in the estimation of esds in distances, angles and torsion angles; correlations between esds in cell parameters are only used when they are defined by crystal symmetry. An approximate (isotropic) treatment of cell esds is used for estimating esds involving l.s. planes.

### Fractional atomic coordinates and isotropic or equivalent isotropic displacement parameters (Å^2^)

**Table:**

	*x*	*y*	*z*	*U*_iso_*/*U*_eq_	Occ. (<1)
Br1	0.20539 (2)	0.04724 (6)	0.54116 (7)	0.03603 (15)	
C1	0.06221 (13)	−0.0182 (5)	0.1208 (6)	0.0249 (7)	
C2	0.10666 (12)	−0.0797 (4)	0.0348 (6)	0.0263 (8)	
H2	0.107547	−0.136872	−0.109346	0.032*	
C3	0.14960 (12)	−0.0586 (5)	0.1562 (6)	0.0269 (8)	
H3	0.179825	−0.098688	0.095236	0.032*	
C4	0.14765 (12)	0.0211 (5)	0.3664 (6)	0.0234 (7)	
C5	0.10402 (12)	0.0822 (4)	0.4573 (6)	0.0241 (7)	
H5	0.103375	0.137253	0.602660	0.029*	
C6	0.06141 (12)	0.0622 (5)	0.3344 (6)	0.0247 (8)	
H6	0.031373	0.103567	0.396099	0.030*	
N1	0.0189 (6)	−0.0524 (16)	−0.013 (3)	0.0249 (7)	0.5
C7	0.0200 (8)	−0.0280 (19)	−0.026 (4)	0.0249 (7)	0.5
H7	0.023969	−0.080904	−0.171946	0.037*	0.5

### Atomic displacement parameters (Å^2^)

**Table:**

	*U*^11^	*U*^22^	*U*^33^	*U*^12^	*U*^13^	*U*^23^
Br1	0.02284 (19)	0.0454 (2)	0.0398 (2)	−0.00407 (19)	−0.00786 (17)	−0.0014 (2)
C1	0.0279 (12)	0.0196 (18)	0.0272 (15)	−0.0043 (13)	−0.0033 (11)	0.0045 (13)
C2	0.0331 (18)	0.0231 (19)	0.0229 (17)	−0.0017 (14)	−0.0001 (15)	−0.0011 (16)
C3	0.0257 (16)	0.028 (2)	0.0267 (19)	0.0032 (17)	0.0051 (14)	−0.0001 (17)
C4	0.0196 (15)	0.0229 (18)	0.0276 (18)	−0.0043 (14)	−0.0001 (13)	0.0045 (15)
C5	0.0287 (17)	0.0206 (19)	0.0229 (16)	−0.0023 (13)	0.0026 (15)	0.0005 (15)
C6	0.0191 (15)	0.0226 (19)	0.032 (2)	−0.0011 (15)	0.0016 (14)	−0.0002 (17)
N1	0.0279 (12)	0.0196 (18)	0.0272 (15)	−0.0043 (13)	−0.0033 (11)	0.0045 (13)
C7	0.0279 (12)	0.0196 (18)	0.0272 (15)	−0.0043 (13)	−0.0033 (11)	0.0045 (13)

### Geometric parameters (Å, º)

**Table:**

Br1—C4	1.900 (3)	C3—H3	0.9500
C1—C6	1.394 (5)	C4—C5	1.385 (5)
C1—C2	1.395 (5)	C5—C6	1.383 (5)
C1—N1	1.448 (17)	C5—H5	0.9500
C1—C7	1.45 (2)	C6—H6	0.9500
C2—C3	1.387 (5)	N1—N1^i^	1.30 (3)
C2—H2	0.9500	C7—C7^i^	1.21 (4)
C3—C4	1.374 (5)	C7—H7	0.9500
			
C6—C1—C2	118.7 (3)	C3—C4—Br1	120.1 (3)
C6—C1—N1	123.8 (8)	C5—C4—Br1	118.4 (3)
C2—C1—N1	117.4 (7)	C6—C5—C4	119.4 (3)
C6—C1—C7	123.6 (9)	C6—C5—H5	120.3
C2—C1—C7	117.5 (9)	C4—C5—H5	120.3
C3—C2—C1	121.1 (3)	C5—C6—C1	120.5 (3)
C3—C2—H2	119.5	C5—C6—H6	119.8
C1—C2—H2	119.5	C1—C6—H6	119.8
C4—C3—C2	118.8 (3)	N1^i^—N1—C1	119.0 (18)
C4—C3—H3	120.6	C7^i^—C7—C1	123 (3)
C2—C3—H3	120.6	C7^i^—C7—H7	118.3
C3—C4—C5	121.5 (3)	C1—C7—H7	118.3
			
C6—C1—C2—C3	−1.3 (5)	C4—C5—C6—C1	−0.1 (5)
N1—C1—C2—C3	−177.6 (5)	C2—C1—C6—C5	0.7 (5)
C7—C1—C2—C3	173.6 (6)	N1—C1—C6—C5	176.7 (6)
C1—C2—C3—C4	1.3 (5)	C7—C1—C6—C5	−173.9 (6)
C2—C3—C4—C5	−0.7 (5)	C6—C1—N1—N1^i^	28.0 (17)
C2—C3—C4—Br1	178.5 (3)	C2—C1—N1—N1^i^	−155.9 (13)
C3—C4—C5—C6	0.1 (5)	C6—C1—C7—C7^i^	−2 (2)
Br1—C4—C5—C6	−179.1 (3)	C2—C1—C7—C7^i^	−177.0 (15)

Symmetry code: (i) −*x*, −*y*, −*z*.

### Hydrogen-bond geometry (Å, º)

*Cg*1 is the centroid of the C1–C6 ring.

**Table:**

*D*—H···*A*	*D*—H	H···*A*	*D*···*A*	*D*—H···*A*
C2—H2···*Cg*1^ii^	0.95	2.81	3.535 (3)	134
C5—H5···*Cg*1^iii^	0.95	2.78	3.494 (3)	133

Symmetry codes: (ii) *x*, −*y*−1/2, *z*−1/2; (iii) *x*, −*y*+1/2, *z*+1/2.
