# Supplementary figures and images for: Synthesis, crystal structure and Hirshfeld surface analysis of the ortho­rhom­bic polymorph of 4-bromo-N-(4-bromo­benzyl­idene)aniline
Source: Acta Crystallogr E Crystallogr Commun. 2023 Feb 9;79(Pt 3):146–50. doi: 10.1107/S2056989023001111 (PMC9993916; doi:10.1107/S2056989023001111)

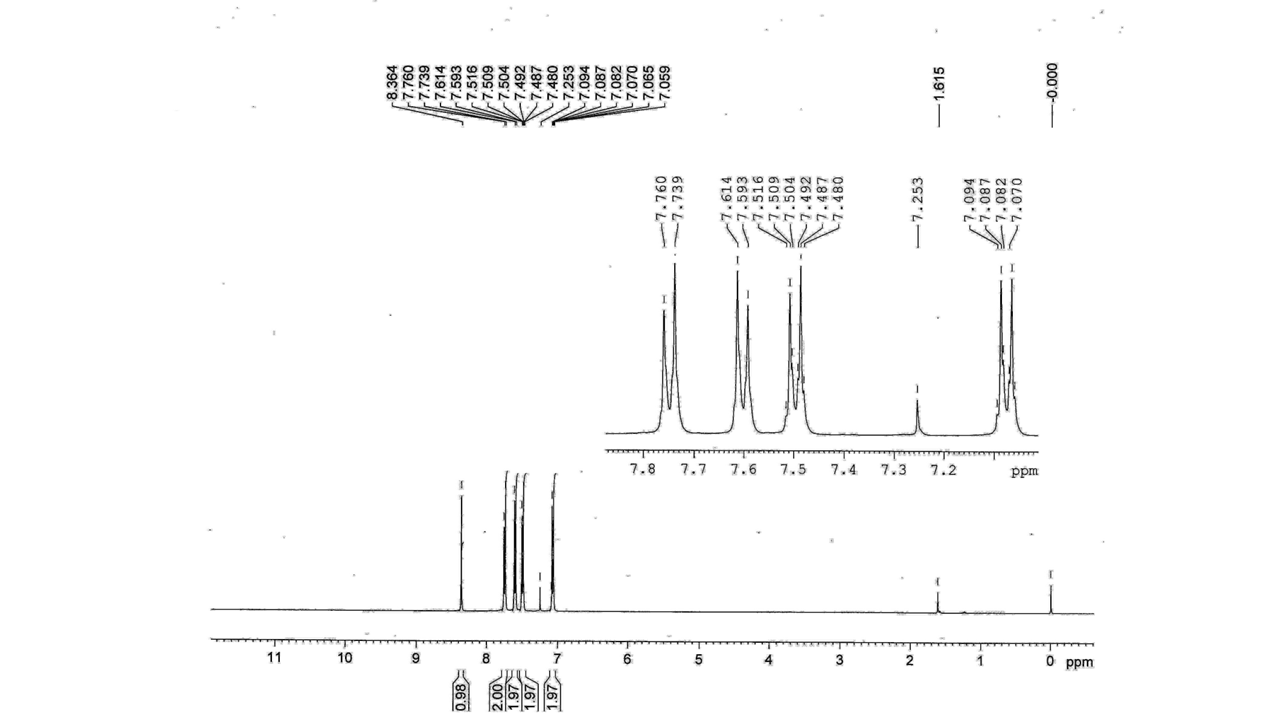

Supplement: Supplementary file 3 [file e-79-00146-sup3.tif]

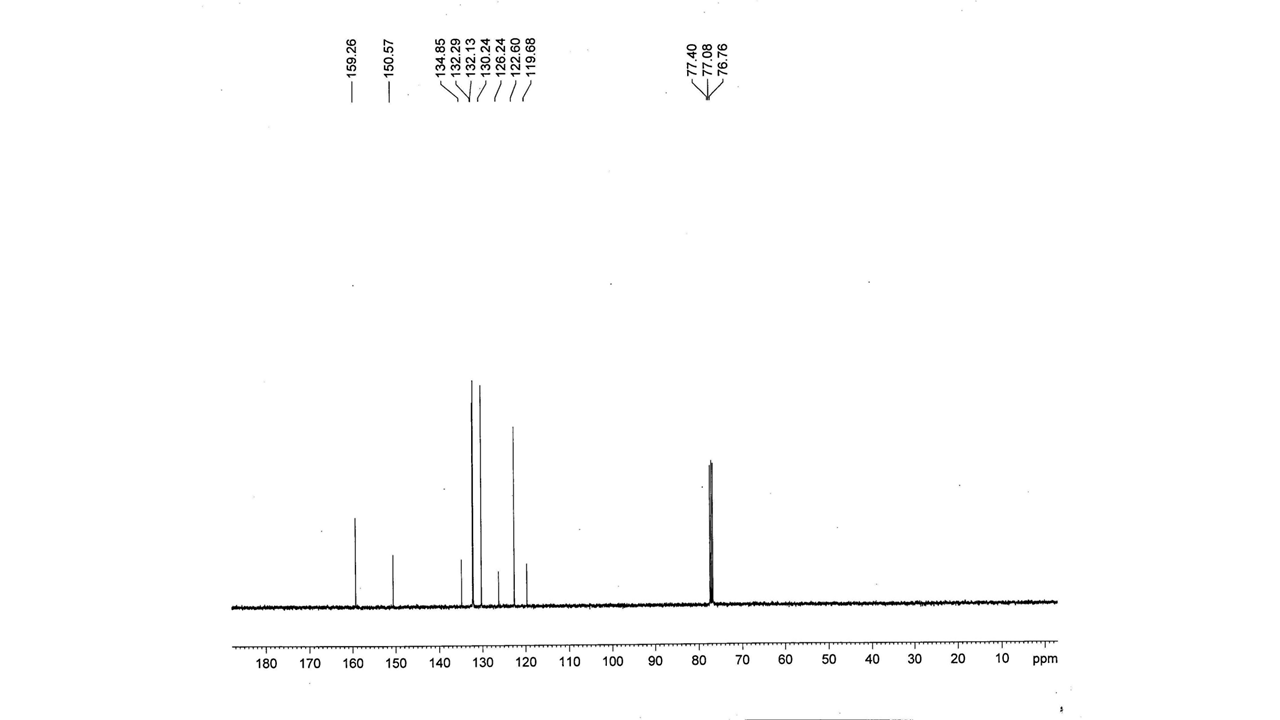

Supplement: Supplementary file 4 [file e-79-00146-sup4.tif]
