# Supplementary material for: Synthesis, crystal structure and Hirshfeld surface analysis of the ortho­rhom­bic polymorph of 4-bromo-N-(4-bromo­benzyl­idene)aniline
Source: Acta Crystallogr E Crystallogr Commun. 2023 Feb 9;79(Pt 3):146–50. doi: 10.1107/S2056989023001111 (PMC9993916; doi:10.1107/S2056989023001111)
Supplement: Supplementary file 5 [file e-79-00146-sup5.pdf]

Data treatment for the *Z*-scan

The best fit for the data was obtained for a two-photon process described by the non-linear transmission equation (**1**) (Sutherland, 1996):

$$T = \left(\frac{(1-R)^2 \exp(-\alpha L)}{\sqrt{\pi q_o}} \right) \int_{-\infty}^{+\infty} In \sqrt{1 + q_o \exp\left(-t^2\right)} dt$$

where T is the intensity dependent transmittance of the sample. L is the length of the sample, and R is its surface reflectivity. α is the linear absorption coefficient. q_0 is given by $\beta(1-R)I_0L_{eff}$, where β is the two-photon absorption coefficient and I_0 is the peak intensity. L_{eff} is given by [1-exp(-aL)]/a. In the present case the quantity β is the effective 2PA coefficient, which has contributions from both resonant and non-resonant nonlinear processes (ESA and 2PA respectively). The resonant term is given by the expression $\beta_{ESA}I = \sigma N$, where σ is the ESA cross-section and N (I) is the intensity-dependent excited states population density.

By determining the best-fit curve for the Z-scan data, the effective two-photon absorption coefficient β is calculated to be 0.13×10^{-10} m W^{-1}. Under similar excitation conditions, N-benzylideneaniline Schiff base NLO materials like 4-bromo-4'-nitrobenzilideneaniline (Subashini *et al.*, 2013b) and 4-bromo-4'-dimethylaminobenzylideneaniline (Subashini *et al.*, 2013*a*) exhibit effective 2PA coefficient values in the range of 10^{-11}–10^{-12} m W^{-1}. An ideal optical limiting material should strongly attenuate intense and potentially dangerous laser beams, while exhibiting high transmittance for low intensity ambient light (Jiang *et al.*, 2011). To characterize the optical limiting performance of the material, energy transmission of the sample was calculated as a function of input fluence. Since a spatially Gaussian laser beam is used, each Z position will correspond to an input laser energy density (fluence), given by eqn. (**2**)

$E(z) = 4(\ln 2)^{1/2}E_{in} / \pi^{3/2}\omega(z)^2$ (**2**)

Where E_{in} is the input laser pulse energy. ω(z) is the beam radius given by eqn. (**3**)

$\omega(z) = \omega(0)[1+(z/z_0)^2]^{1/2}$ (**3**)

where ω(0) is the beam radius at the focus and $z_0 = \pi\omega(0)^2/l$ is the Rayleigh range (diffraction length). l is the excitation wavelength.

Fig. S3 shows the optical limiting curve of the sample, which is calculated from the Z-scan data using equations **2** and **3**. Thus, the sample of Form II shows optical limiting behaviour.

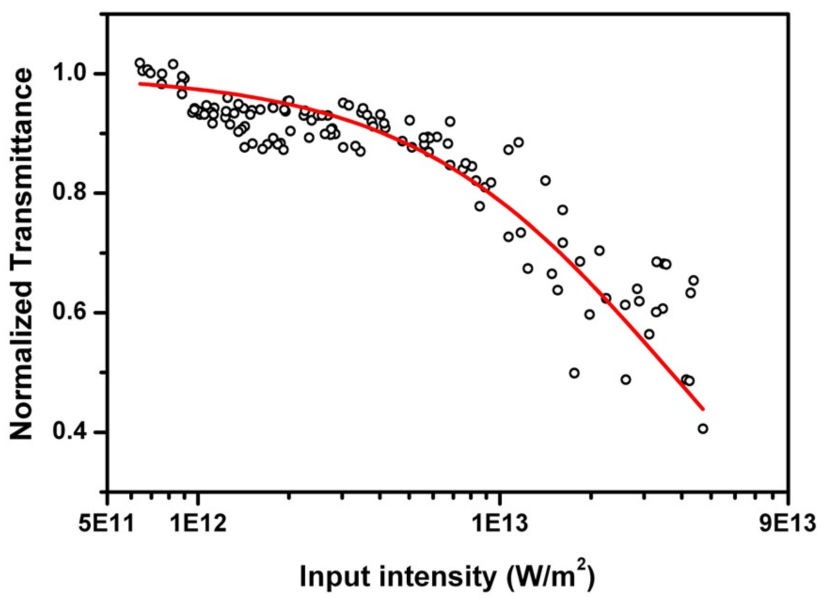

References

Jiang, Y., Wang, Y., Yang, J., Hua, J., Wang, B., Qian, S. & Tia, H. (2011). *J. Poly. Sci. A: Poly. Chem*., 49, 1830–1839.

Sutherland, R. L. (1996). *Handbook of Nonlinear Optics*. Marcel Dekker Inc., Germany.
